# Supplementary material for: Structure-based design of chimeric antigens for multivalent protein vaccines
Source: Nat Commun. 2018 Mar 13;9:1051. doi: 10.1038/s41467-018-03146-7 (PMC5849709; doi:10.1038/s41467-018-03146-7)
Supplement: Supplementary file 1 — Supplementary Information [file 41467_2018_3146_MOESM1_ESM.pdf]

Structure-based design of chimeric antigens for multivalent protein vaccines

Hollingshead et al.

Supplementary Information

Supplementary Figure 1. Differential scanning calorimetry.

Supplementary Figure 2. Structural alignments of ChAs with wild type fHbp.

Supplementary Figure 3. Surface plasmon resonance of $CFH_{6/7}$ interacting with ChAs.

Supplementary Figure 4. Flow cytometry histograms showing detection of fHbp and PorA by ChA antisera.

Supplementary Figure 5. Complete gel and Western blots from Figure 1C.

Supplementary Figure 6. Complete gel and Western blots from Figure 2B.

Supplementary Figure 7. Complete gel and Western blots from Figure 2D.

Supplementary Figure 8. Complete gel and Western blots from Figure 4E.

Supplementary Figure 9. Complete gel and Western blots from Figure 4F.

Supplementary Table 1: ChA nomenclature

Supplementary Table 2: *Neisseria meningitidis* strains

Supplementary Table 3: *Escherchia coli* strains

Supplementary Table 4: Primers

Supplementary Table 5: Data collection and refinement statistics.

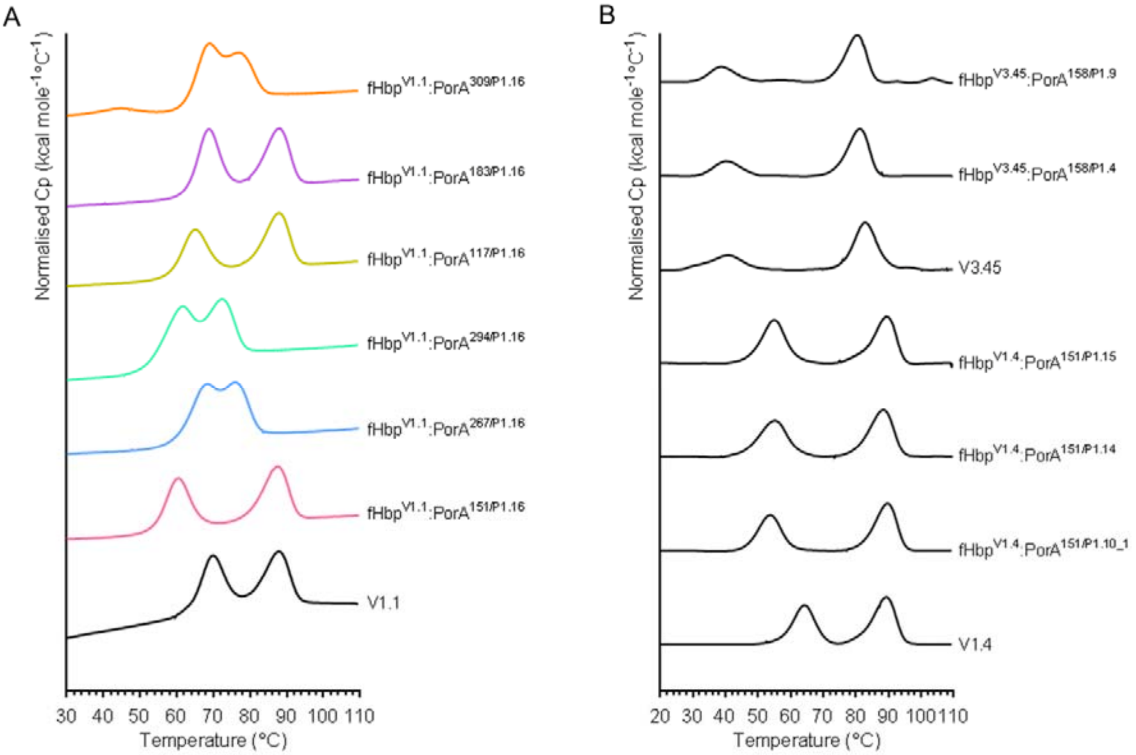

Supplementary Figure 1. Differential scanning calorimetry.

Normalised thermal melt traces of ChAs composed of (**A**) fHbp V1.1 and PorA VR2 P1.16, or (**B**) fHbp V1.4 and VR2 peptides P1.10_1, P1.14 and P1.15 inserted at position 151, or fHbp V3.45 and VR2 peptides P1.4 or P1.9 inserted at position 158.

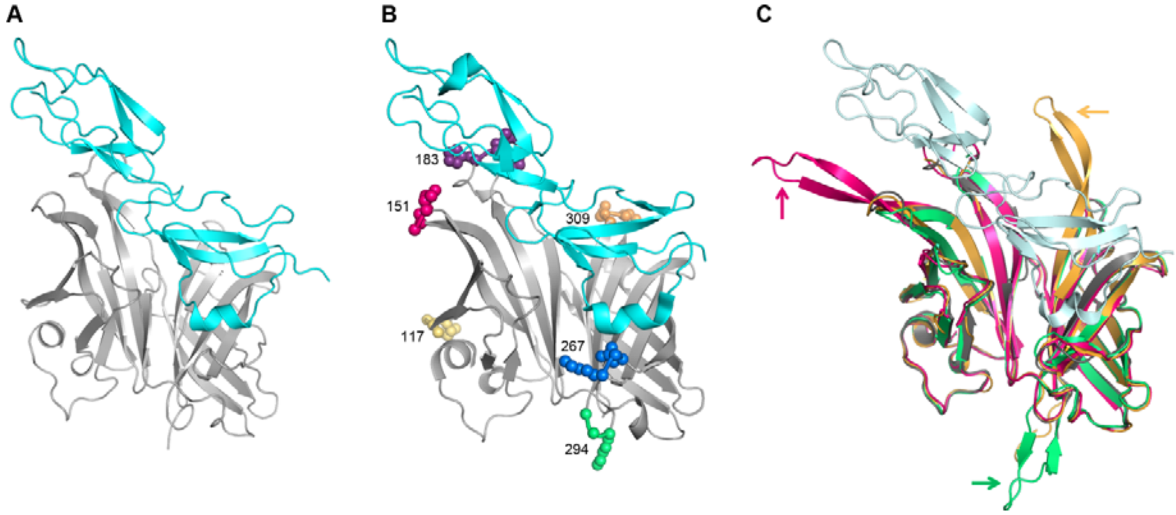

Supplementary Figure 2. Structural alignments of ChAs with wild type fHbp.

(**A**) Complex formed by V1.1 fHbp (grey) and $CFH_{6/7}$ (cyan, PDB ID 2W80). (**B**) Locations of the six fHbp residues replaced with VR2 P1.16 in relation to the $CFH_{6/7}$. (**C**) Secondary structure alignment of ChAs with fHbp V1.1 (grey) in a complex with $CFH_{6/7}$ (light blue); $fHbp^{V1.4}$:$PorA^{151/P1.16}$ (pink), $fHbp^{V1.1}$:$PorA^{294/P1.16}$ (green), $fHbp^{V1.4}$:$PorA^{309/P1.16}$ (orange), VR2 P1.16 is indicated by the correspondingly coloured arrows.

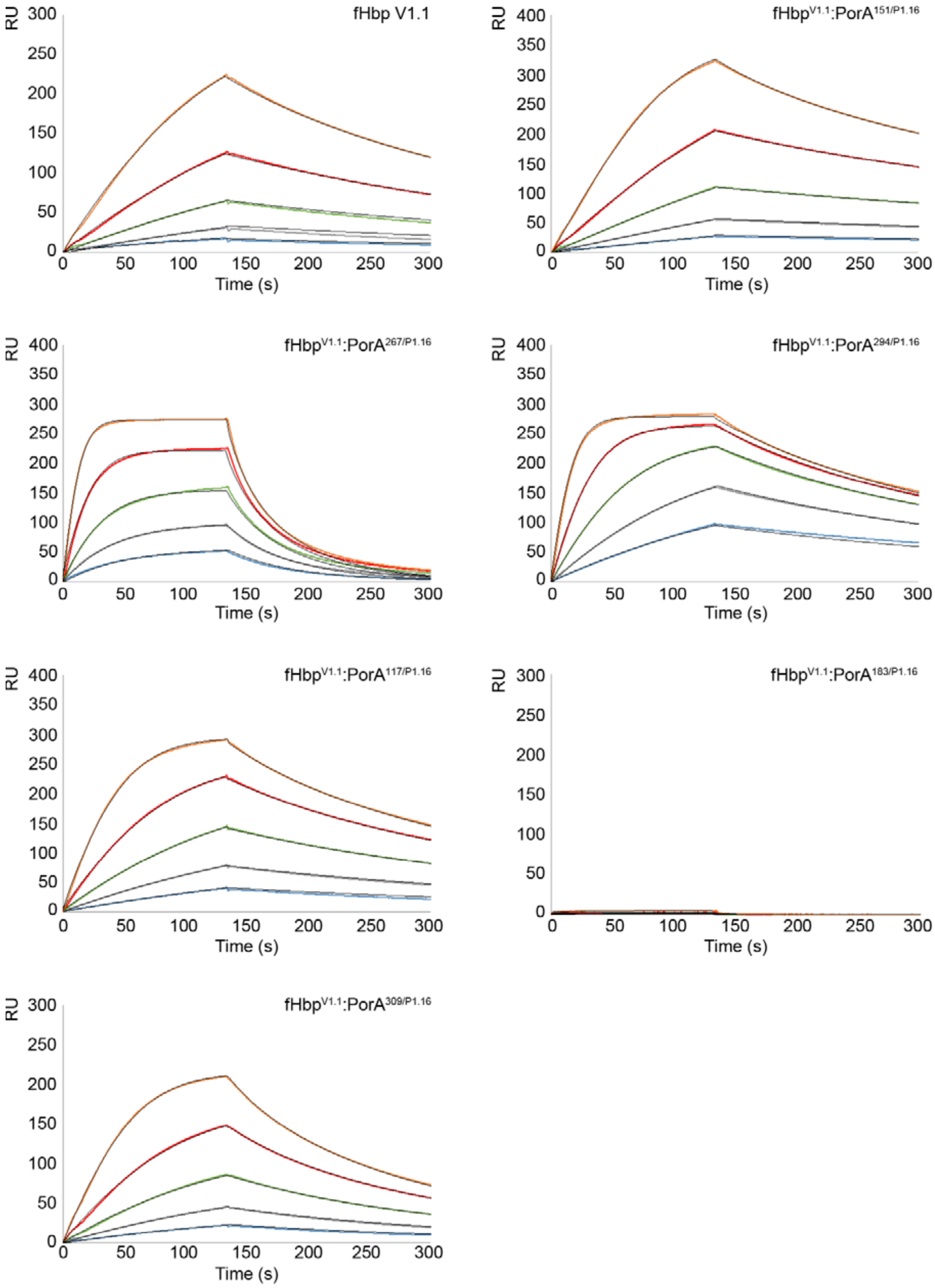

Supplementary Figure 3. Surface plasmon resonance of CFH$_{6/7}$ interacting with ChAs.

The ChA was immobilized on the chip surface and increasing concentrations of CFH[67] flowed over the surface: 1nM (blue), 2nM (grey), 4nM (green), 8nM (red), 16nM (orange). Binding was analyzed using the BiaEvaluation software and the fits to the curves are shown as black lines. Kd values are reported in **Table 1**.

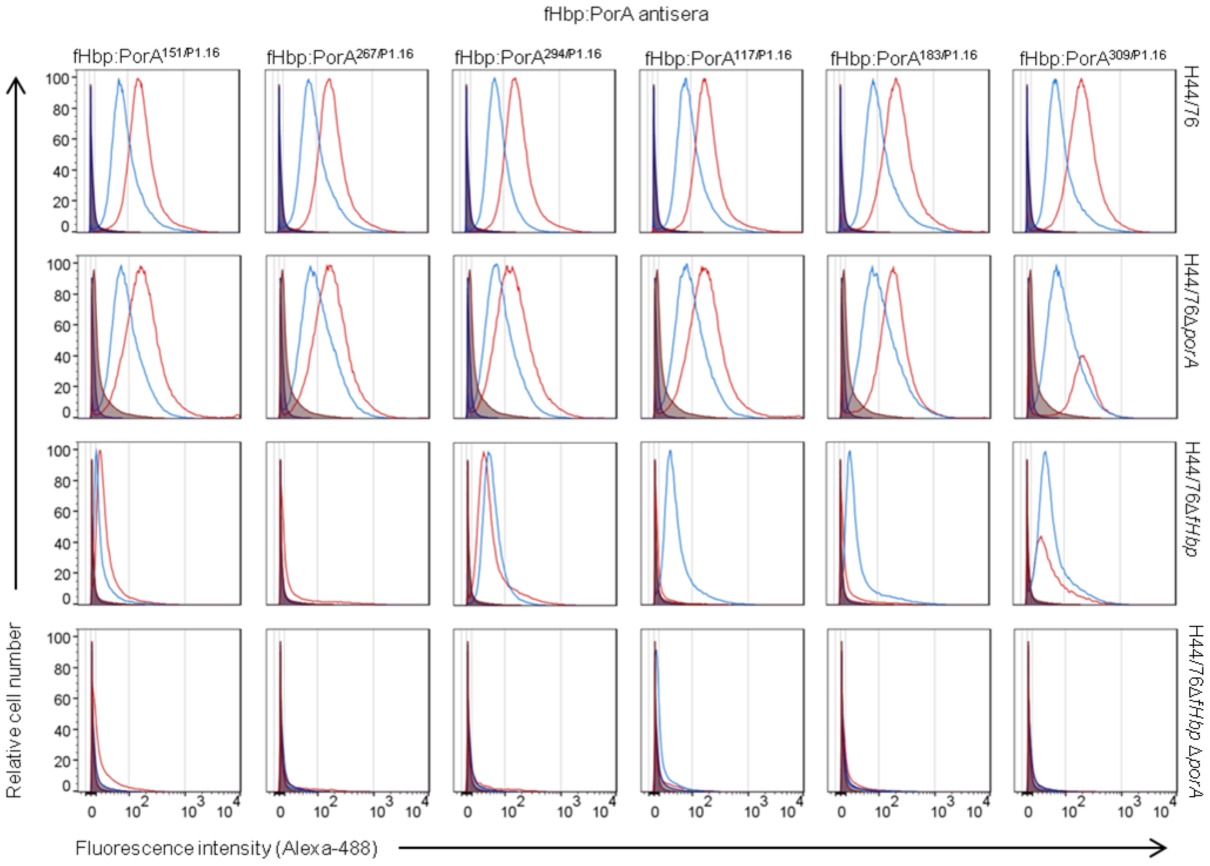

Supplementary Figure 4. **Flow cytometry histograms showing detection of fHbp and PorA by ChA antisera.** Histograms of flow cytometry experiments conducted with ChA/alum (blue) and ChA/MPLA (red) antisera binding to fixed *N. meningitidis* H44/76 strains: wild type, Δ*fHbp,* Δ*porA* and Δ*fHbp*Δ*porA*. fHbp and PorA antibodies bound to *N. meningitidis* were detected using Alexa-488 conjugated IgA/G/M secondary antibody. Alum PBS and MPLA PBS antisera are shown in dark blue and dark red shaded profiles, and secondary IgAGM control is shown in black. Comparisons of the geometric means from these histograms, and the histograms from two other independent repeats, are shown in **Figures 2C** and **2D**.

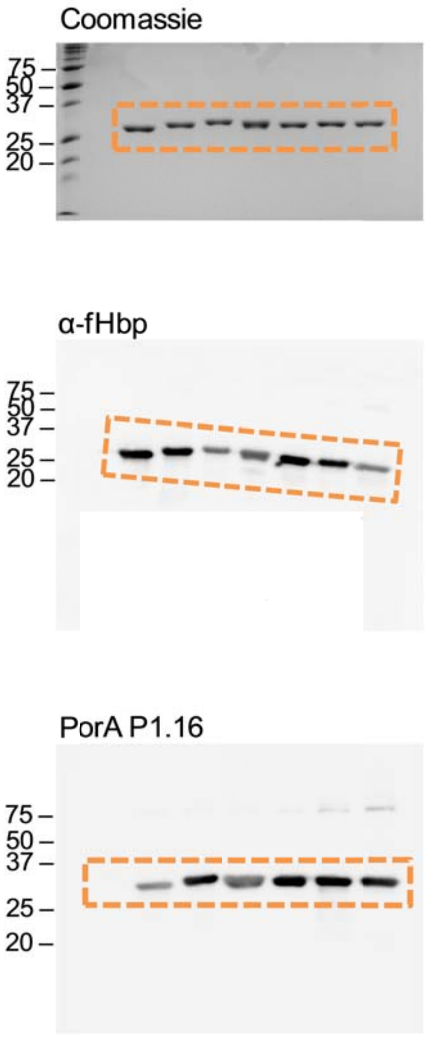

Supplementary Figure 5. Complete gel and Western blots from Figure 1C.

0

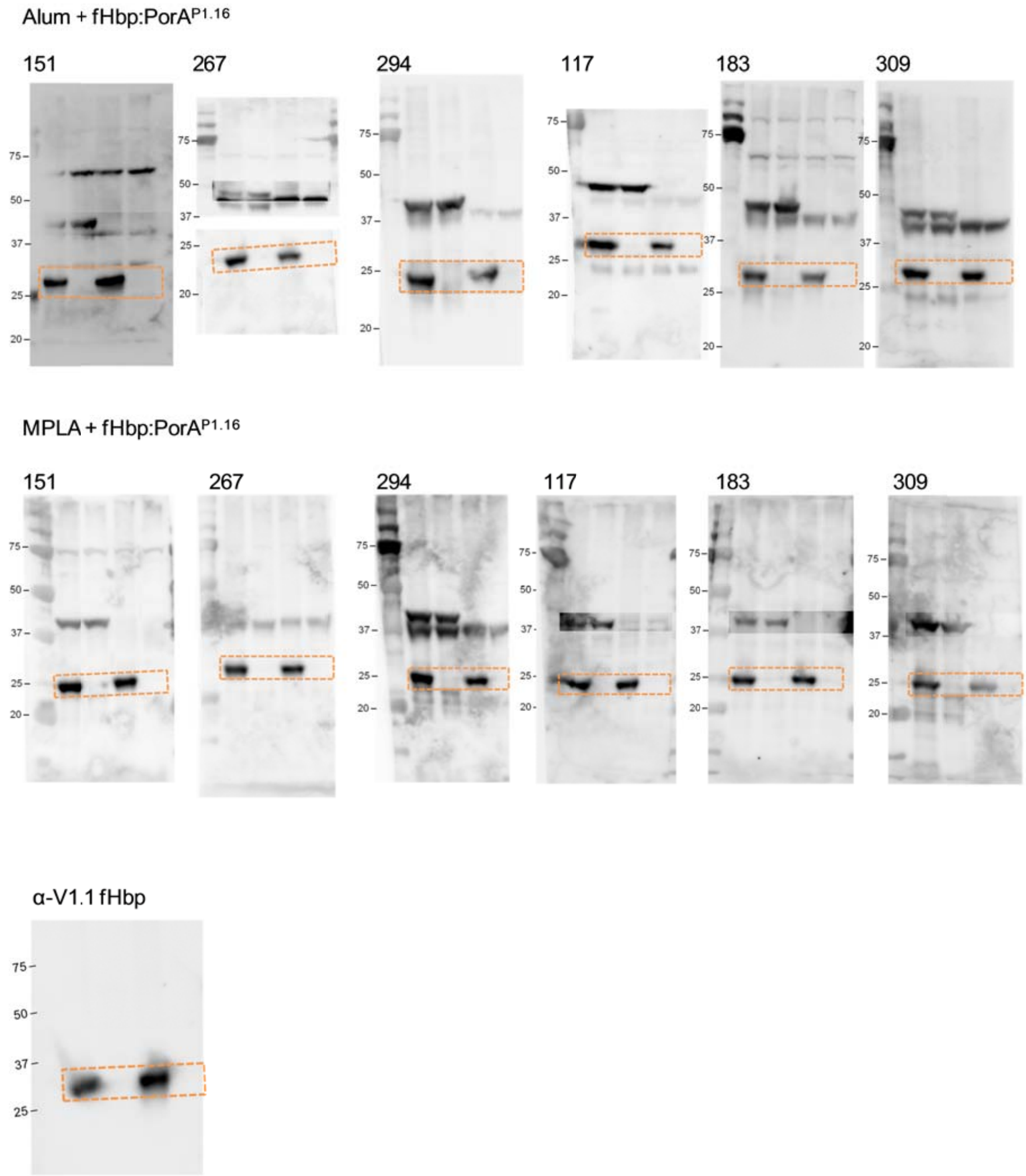

Supplementary Figure 6. Complete Western blots from Figure 2B.

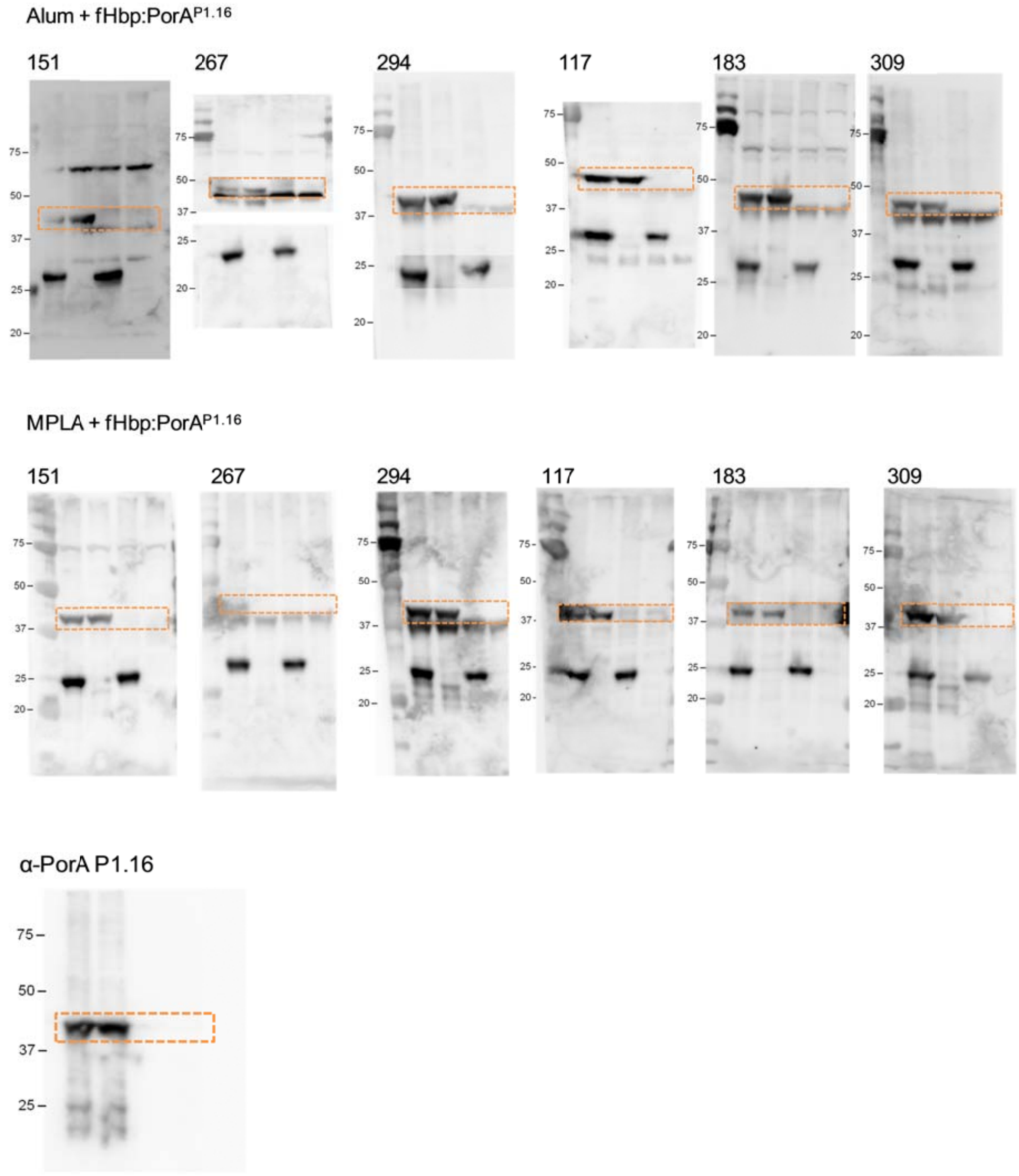

Supplementary Figure 7. Complete Western blots from Figure 2D.

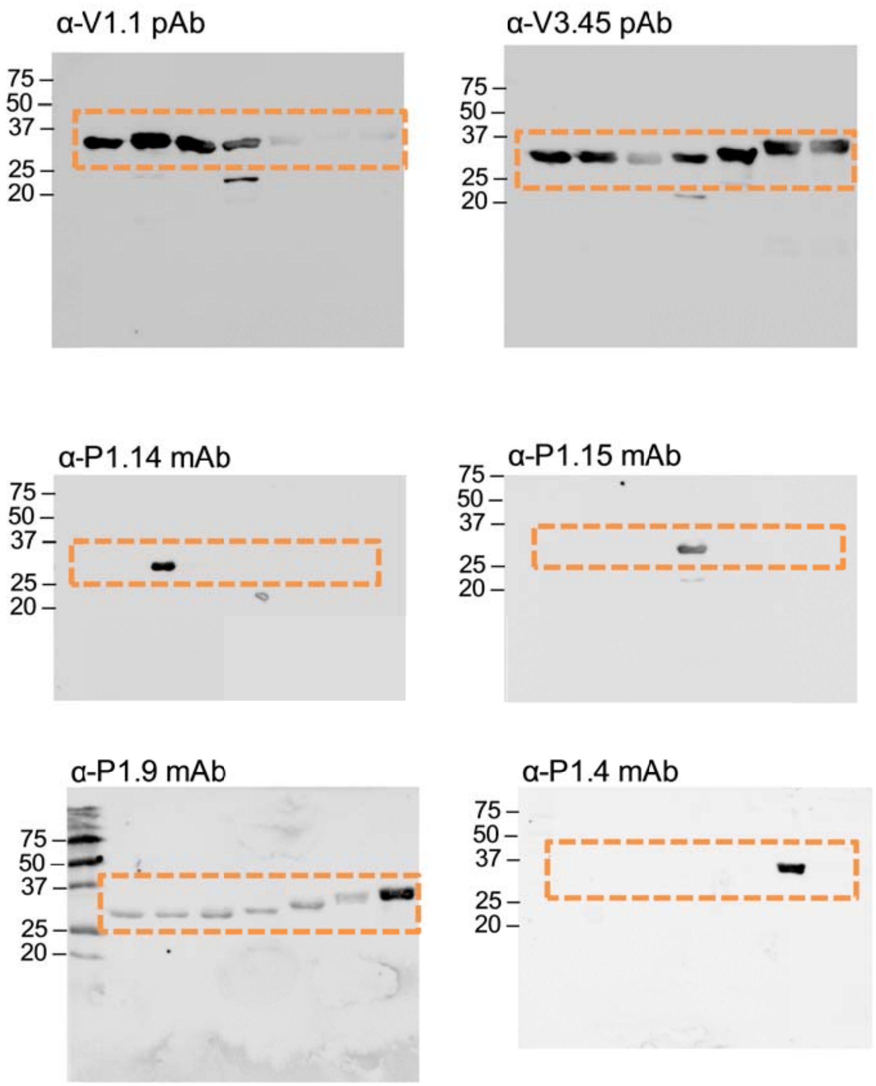

Supplementary Figure 8. Complete Western blots from Figure 4E.

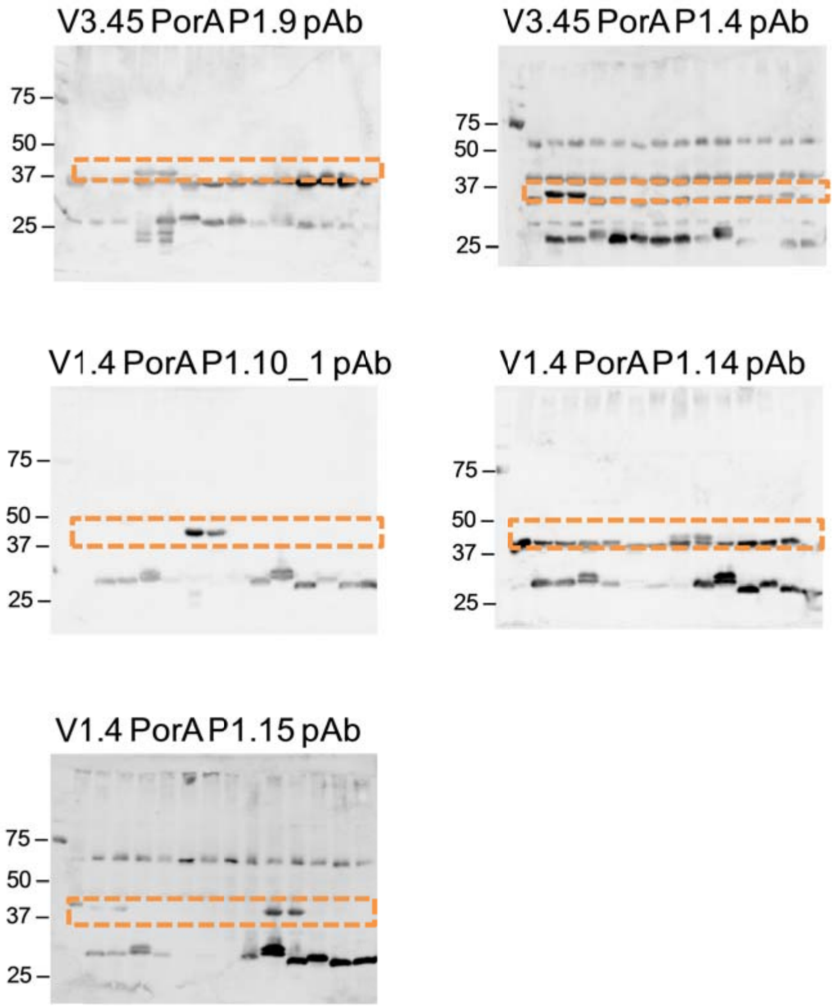

Supplementary Figure 9. Complete Western blots from Figure 4F

Supplementary Table 1: ChA nomenclature

fHbp scaffold	PorA VR2 loop inserted	Residue position of PorA VR2 loop insertion	ChA
V1.1	P1.16	151	$fHbp^{V1.1}$:$PorA^{151/P1.16}$
V1.1	P1.16	267	$fHbp^{V1.1}$:$PorA^{267/P1.16}$
V1.1	P1.16	294	$fHbp^{V1.1}$:$PorA^{294/P1.16}$
V1.1	P1.16	117	$fHbp^{V1.1}$:$PorA^{117/P1.16}$
V1.1	P1.16	183	$fHbp^{V1.1}$:$PorA^{183/P1.16}$
V1.1	P1.16	309	$fHbp^{V1.1}$:$PorA^{309/P1.16}$
V1.4	P1.16	151	$fHbp^{V1.4}$:$PorA^{151/P1.16}$
V1.4	P1.16	309	$fHbp^{V1.4}$:$PorA^{309/P1.16}$
V1.4	P1.10_1	151	$fHbp^{V1.4}$:$PorA^{151/P1.1.10\_1}$
V1.4	P1.14	151	$fHbp^{V1.4}$:$PorA^{151/P1.14}$
V1.4	P1.15	151	$fHbp^{V1.4}$:$PorA^{151/P1.15}$
V3.45	P1.4	158	$fHbp^{V3.45}$:$PorA^{158/P1.4}$
V3.45	P1.9	158	$fHbp^{V3.45}$:$PorA^{158/P1.9}$

Supplementary Table 2: ***Neisseria meningitidis* strains**

Strain	fHbp	PorA VR1	PorA VR2	Year	Serogroup	Source
H44/76	V1.1	P1.7	P1.16	1976	B	[80]
H44/76ΔfHbp	deleted	P1.7	P1.16	1976	B	
H44/76ΔPorA	V1.1	P1.7	deleted	1976	B	This study
H44/76ΔfHbp ΔPorA	deleted	P1.7	deleted	1976	B	
M10.0240129	V1.92*	P1.7_2	P1.4	2010	B	Meningococcal Reference Unit. Public Health England
M10.0240123	V1.92*	P1.7_2	P1.4	2010	B	
M11 240428	V1.13	P1.22	P1.9	2011	B	
M11 240431	V2.19	P1.22	P1.9	2011	B	
M11 240189	V3.84	P1.5_1	P1.10_1	2011	B	
M11 240287	V2.584	P1.5_1	P1.10_1	2011	B	
M15 240917	V3.45	P1.22	P1.14	2015	B	
M15 240923	V1.4	P1.18_1	P1.14	2015	B	
M07 240902	V1.220	P1.19	P1.15	2007	B	
M08 240404	V1.1	P1.19	P1.15	2008	B	
M11 240382	V1.4	P1.12_1	P1.16	2011	B	
M11 240406	V1.1	P1.7	P1.16	2011	B	

*fHbp truncated at residue 242

Supplementary Table 3: *Escherchia coli* strains

Strain	Plasmid	Protein expressed
B834	pET21b-fHbp$^{V1.1}$	V1.1 fHbp
B834	pET21b-fHbp$^{V1.1}$:PorA$^{151/P1.16}$	fHbp$^{V1.1}$:PorA$^{151/P1.16}$
B834	pET21b-fHbp$^{V1.1}$:PorA$^{267/P1.16}$	fHbp$^{V1.1}$:PorA$^{267/P1.16}$
B834	pET21b-fHbp$^{V1.1}$:PorA$^{294/P1.16}$	fHbp$^{V1.1}$:PorA$^{294/P1.16}$
B834	pET21b-fHbp$^{V1.1}$:PorA$^{117/P1.16}$	fHbp$^{V1.1}$:PorA$^{117/P1.16}$
B834	pET21b-fHbp$^{V1.1}$:PorA$^{183/P1.16}$	fHbp$^{V1.1}$:PorA$^{183/P1.16}$
B834	pET21b-fHbp$^{V1.1}$:PorA$^{309/P1.16}$	fHbp$^{V1.1}$:PorA$^{309/P1.16}$
B834	pET21b-fHbp$^{V1.4}$:PorA$^{151/P1.16}$	fHbp$^{V1.4}$:PorA$^{151/P1.16}$
B834	pET21b-fHbp$^{V1.4}$:PorA$^{309/P1.16}$	fHbp$^{V1.4}$:PorA$^{309/P1.16}$
B834	pET21b-fHbp$^{V1.4}$:PorA$^{151/P1.1.10_1}$	fHbp$^{V1.4}$:PorA$^{151/P1.1.10_1}$
B834	pET21b-fHbp$^{V1.4}$:PorA$^{151/P1.14}$	fHbp$^{V1.4}$:PorA$^{151/P1.14}$
B834	pET21b-fHbp$^{V1.4}$:PorA$^{151/P1.15}$	fHbp$^{V1.4}$:PorA$^{151/P1.15}$
B834	pET21b-fHbp$^{V3.45}$:PorA$^{158/P1.4}$	fHbp$^{V3.45}$:PorA$^{158/P1.4}$
B834	pET21b-fHbp$^{V3.45}$:PorA$^{158/P1.9}$	fHbp$^{V3.45}$:PorA$^{158/P1.9}$

Supplementary Table 4: Primers

Primer Name	Sequence
fHbp F1	GCGCATATGGTCGCCGCCGACATCG
fHbp R4	GCGCTCGAGTTGCTTGGCAGCAAGACCGATATGG
fHbp$^{V1.1}$:PorA$^{151/P1.16}$ R2	CGCCAAATCGAAGTGGACTACTACACCAAAGACACCAACAACAACCTGACCCTGGTCCAGCTCATTACCTTGGAG
fHbp$^{V1.1}$:PorA$^{151/P1.16}$ F3	CTCCAAGGTAATGAGCTGGACCAGGGTCAGGTTGTTGTTGGTGTCTTTGGTGTAGTAGTCCACTTCGATTTGGCG
fHbp$^{V1.1}$:PorA$^{267/P1.16}$ R2	AAGCCGGATTACTACACCAAAGACACCAACAACAACCTGACCCTGGTCAAACGCCAT
fHbp$^{V1.1}$:PorA$^{267/P1.16}$ F3	ATGGCGTTTGACCAGGGTCAGGTTGTTGTTGGTGTCTTTGGTGTAGTAATCCGGCTT
fHbp$^{V1.1}$:PorA$^{294/P1.16}$ R2	TACTACACCAAAGACACCAACAACAACCTGACCCTGGTCAAAGCCCAGGAA
fHbp$^{V1.1}$:PorA$^{294/P1.16}$ F3	GACCAGGGTCAGGTTGTTGTTGGTGTCTTTGGTGTAGTAGCCAAAGATACC
fHbp$^{V1.1}$:PorA$^{117/P1.16}$ R2	GACCAGGGTCAGGTTGTTGTTGGTGTCTTTGGTGTAGTATTGTGCCGCCAGCTTCAGCTTT
fHbp$^{V1.1}$:PorA$^{117/P1.16}$ F3	TACTACACCAAAGACACCAACAACAACCTGACCCTGGTCGCGGAAAAAACTTATGGAAACGGTGAC
fHbp$^{V1.1}$:PorA$^{183/P1.16}$ R2	GACCAGGGTCAGGTTGTTGTTGGTGTCTTTGGTGTAGTACGAATCTTGTATTTGCTCGGTCTGAAAGG
fHbp$^{V1.1}$:PorA$^{183/P1.16}$ F3	TACTACACCAAAGACACCAACAACAACCTGACCCTGGTCCATTCCGGGAAGATGGTTGCG
fHbp$^{V1.1}$:PorA$^{309/P1.16}$ R2	GACCAGGGTCAGGTTGTTGTTGGTGTCTTTGGTGTAGTATACGGTTTTCACTTCCGCGCTGC
fHbp$^{V1.1}$:PorA$^{309/P1.16}$ R4	GTTCTCGAGTTGCTTGGCAGCAAGACCGATATGGCGTATGCCGACCAGGGTCAGGTTGTTGTTGG
fHbp$^{V1.4}$:PorA$^{151/P1.16}$ R1	CGGGACCAGCGTCAGGTTGTTATTTGTATCCTTGGTATAGTAGTCCACTTCGATTTGACGGATAAAGTCG
fHbp$^{V1.4}$:PorA$^{151/P1.16}$ F2	TACTATACCAAGGATACAAATAACAACCTGACGCTGGTCCCGCAGCTCATTACCTTGGAGAGCGGAG
fHbp$^{V1.4}$:PorA$^{309/P1.16}$ R1	CGGGACCAGCGTCAGGTTGTTATTTGTATCCTTGGTATAGTATGCGGTTTCCACTTCTGCGCTGC
fHbp$^{V1.4}$:PorA$^{309/P1.16}$ R2	ATACTCGAGTTGCTTTGCAGCAAGACCAATATGTCGTATTCCCGGGACCAGCGTCAGG
fHbp$^{V1.4}$:PorA$^{151/P1.1.10_1}$ R1	CGGCACCAGGGTTGGCGGTTGGTTCTGTTTATTCTGGTCCACTTCGATTTGACGGATAAAGTCG
fHbp$^{V1.4}$:PorA$^{151/P1.1.10_1}$ F2	CAGAATAAACAGAACCAACCGCCAACCCTGGTGCCGCAGCTCATTACCTTGGAGAGCGGAG
fHbp$^{V1.4}$:PorA$^{151/P1.14}$ R1	AGCGTGAACCATTTTCTTTTCATCCACATAGTCCACTTCGATTTGACGGATAAAGTCG
fHbp$^{V1.4}$:PorA$^{151/P1.14}$ F2	TATGTGGATGAAAAGAAAATGGTTCACGCTCAGCTCATTACCTTGGAGAGCGGAG
fHbp$^{V1.4}$:PorA$^{151/P1.15}$ R1	CGGTACAAAAACATCTGCGTTATTTTGACGGGTATAATGGTCCACTTCGATTTGACGGATAAAGTCG
fHbp$^{V1.4}$:PorA$^{151/P1.15}$ F2	CATTATACCCGTCAAAATAACGCAGATGTTTTTGTACCGCAGCTCATTACCTTGGAGAGCGGAG
fHbp$^{V3.45}$:PorA$^{158/P1.4}$ R1	CGGTACATGGGTTGCCACTTTATTATTCACAACAACATGGTCCACTTCGATTTTTTGCACAAAGTCG
fHbp$^{V3.45}$:PorA$^{158/P1.4}$ F2	CATGTTGTTGTGAATAATAAAGTGGCAACCCATGTACCGCAAACCATCACGCTGGCAAGC
fHbp$^{V3.45}$:PorA$^{158/P1.9}$ R1	TGCGTGGTATTTAGACTGTTCGTCTACGTAGTCCACTTCGATTTTTTGCACAAAGTCG
fHbp$^{V3.45}$:PorA$^{158/P1.9}$ F2	TACGTAGACGAACAGTCTAAATACCACGCACAAACCATCACGCTGGCAAGC

PorA KO F1	GCTTCAGCACCTTAGAGAATCG
PorA KO R1	CGCTATCAGGATCCAATCACTAGTGCTGGTAGTTCCTGCCTTCC
PorA KO F2	GGAGATAGTTATTATAAACTACAAGTGGATATTGTGGGACGACCGAA ATTGCCGCCAC
PorA KO R2	CGTGGGAATGACAATTCGAGACCT
PorA(tet) KO F1	CCAGGACATATGGATTTCTTAGCAGAAGTA
PorA(tet) KO R1	GGCTCTTTTAGTTCTATCTCCACATGATACT

Supplementary Table 5: Data collection and refinement statistics.

ChA	fHbp[V1.4] PorA[151/P1.16]	fHbp[V1.1] PorA[294/P1.16]	fHbp[V1.4] PorA[309/P1.16]	fHbp[V1.1] PorA[309/P1.16]
**PDB Code**	**5nqp**	**5nqx**	**5nqy**	**5nqz**
**Data collection statistics**				
Beamline	Diamond I02	Diamond I02	ESRF ID29	Diamond I04-1
Wavelength (Å)	0.97949	0.97858	0.97623	0.92818
Space group	$C222_1$	$P22_12_1$	$P2_12_12_1$	$C2$
Resolution limits (Å)[a]	40.74-2.86 (2.93-2.86)	81.65-3.66 (3.76-3.66)	51.17-2.60 (2.67-2.60)	58.55-1.63 (1.67-1.63)
Unit cell dimensions (Å,°)	100.7,171.4,131.5 90,90,90	94.6,114.9,160.3 90,90,90	56.9,62.6,88.7 90,90,90	91.4,76.3,83.9 90,91.3,90
Unique reflections	26403 (1923)	19820 (1431)	10208 (743)	70772 (4967)
Multiplicity[a]	4.5 (4.4)	4.4 (4.6)	6.4 (6.6)	3.0 (2.2)
Completeness (%)[a]	99.1 (99.4)	99.3 (99.9)	99.6 (99.4)	98.6 (93.9)
$I/\sigma(I)$[a]	17.1 (2.0)	7.6 (1.9)	16.7 (2.3)	12.7 (1.5)
R_{merge} (%)[a,b]	0.074 (0.645)	0.187 (0.833)	0.073 (0.845)	0.062 (0.615)
R_{pim} (%)	0.038 (0.340)	0.101 (0.439)	0.032 (0.356)	0.041 (0.534)
**Refinement statistics**				
Resolution limits (Å)	40.74-2.86 (2.97-2.86)	81.65-3.66 (3.85-3.66)	51.17-2.60 (2.86-2.60)	58.55-1.63 (1.67-1.63)
Number of reflections in working set	25137 (2746)	18807 (2650)	9665 (2369)	63880 (4735)

Number of reflections in test set	1238 (134)	975 (121)	498 (120)	3445 (225)
R factor of working set[a,c]	0.198 (0.303)	0.232 (0.321)	0.226 (0.351)	0.162 (0.289)
R_{free}[a,d]	0.239 (0.351)	0.282 (0.368)	0.271 (0.420)	0.194 (0.297)
Number of atoms (protein/water/other)	5895/28/84	9572/0/0	1994/4/4	4046/540/45
Residues in Ramachandran favoured region (%)	95.2	97.8	95.3	97.0
Ramachandran outliers (%)	0.1	0.1	0.0	0.2
r.m.s.d. bond lengths (Å)	0.002	0.002	0.002	0.020
r.m.s.d. bond angles (°)	0.58	0.42	0.45	1.98

[a]Numbers in parentheses refer to the appropriate outer shell.

[b]R_{merge} = 100 x (Σ_{hkl} Σ_i|*I(hkl;i)* – <*I(hkl)*>|/Σ_{hkl} Σ_i*I(hkl;i))*, where *I(hkl;i)* is the intensity of an individual measurement of a reflection and <*I(hkl)*> is the average intensity of that reflection.

[c]R_{factor} = (Σ_{hkl}||F_{obs}| –|F_{calc}||/Σ_{hkl} |F_{obs}|), where |F_{obs}| and |F_{calc}| are the observed and calculated structure facture amplitudes.

[d]R_{free} equals the *R*-factor of test set (5% of the data removed prior to refinement).

r.m.s.d.: root mean square deviation from ideal geometry.
